# Advances in the mechanism and therapeutic potential of neutrophil extracellular traps in cancer promotion

**DOI:** 10.3389/fonc.2025.1593510

**Published:** 2025-09-22

**Authors:** Xiaorui Li, Huilin Wang, Jingchang Li, Chuangchuang Li, Shuo Zhao, Qing Wang, Weihong Ren

**Affiliations:** ^1^ Department of Clinical Laboratory, The First Affiliated Hospital of Henan University of Chinese Medicine, Zhengzhou, China; ^2^ The First Clinical Medical College, Henan University of Chinese Medicine, Zhengzhou, China

**Keywords:** neutrophils, extracellular traps, promote cancer, tumor immunity, targeted therapy

## Abstract

Neutrophil extracellular traps (NETs) are chromatin-based structures containing histones and granular proteases released during NETosis. They constitute a key antimicrobial defense mechanism while exposing pathogenic histones. While NET components effectively eliminate microorganisms, their pro-inflammatory and cytotoxic properties inflict significant damage on host endothelial cells and tissues. This damage contributes to diverse pathologies, including autoimmune diseases where NET-derived components act as autoantigens, as well as circulatory disorders, diabetes, and especially, cancer. Recent research has increasingly illuminated the critical connection between NETs and cancer progression, highlighting their role in promoting tumor development across all stages through inflammation and tissue injury. Consequently, targeting NET composition, formation, or release has emerged as a promising therapeutic strategy. These approaches effectively mitigate NET-mediated pathogenesis while circumventing the drawbacks of direct neutrophil depletion. Although translating these strategies into widespread clinical practice presents challenges, experimental studies demonstrate significant potential. This review examines the mechanisms by which NETs drive cancer, explores current therapeutic applications targeting NETs, and discusses both the prospects and challenges of this evolving anticancer approach.

## 1 Introduction

Neutrophils serve as critical effector cells in immune defense, employing diverse antimicrobial strategies—phagocytosis, degranulation, reactive oxygen species (ROS) production, and NET formation. These web-like structures—composed of DNA, histones, and granular proteins—enable extracellular pathogen containment. Although spontaneous DNA release from lymphocytes was documented in 1975 (1), the functional significance of extracellular chromatin remained unclear until Brinkmann and colleagues formally defined NETosis in 2004 as a programmed cell death mechanism through which neutrophils expel NETs to capture and kill extracellular bacteria—a concept now widely established (2). Historically viewed as short-lived terminal effector cells (circulating ~12 hours), neutrophils were mischaracterized by an oversimplified perspective hindering recognition of their roles in chronic pathologies that hindered recognition. Beyond acute infection control, NETosis can be triggered by persistent inflammatory stimuli. During chronic inflammation, sustained NET release exacerbates tissue damage and drives disease progression as neutrophils deplete alternative regulatory mechanisms (3). Crucially, neutrophils are now recognized as major constituents of the tumor microenvironment (TME), where they exhibiting context-dependent functions across all cancer stages. While protumor roles dominate current literature, NETs specifically have emerged as key mediators of cancer initiation and progression—facilitating DNA damage, metastatic dissemination, and inflammatory cascades within the TME.

**Figure 1:**
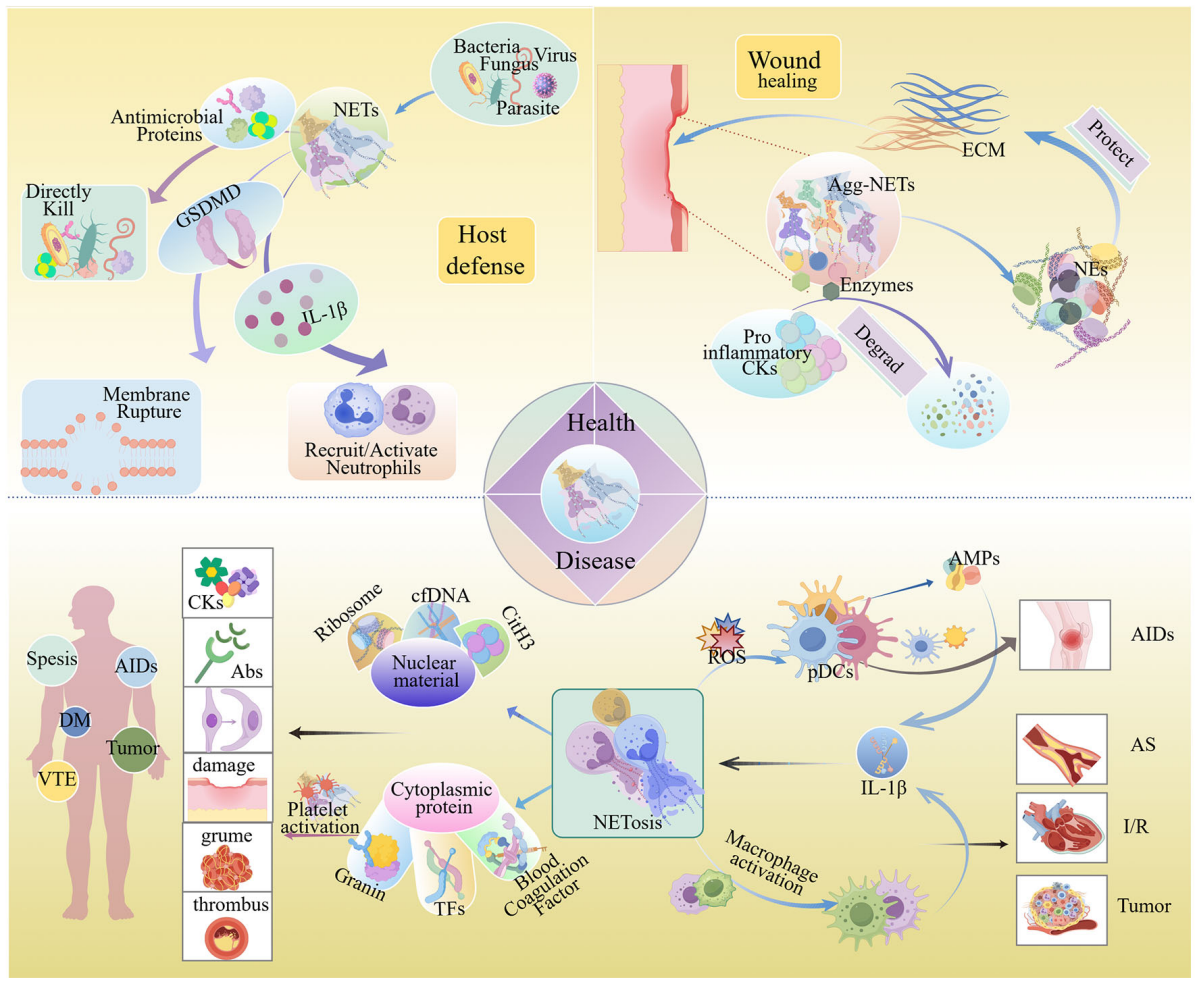
NETs in physiological and pathological conditions. Under physiological conditions, NETs exert host defense effects through directly killing pathogens, causing pathogen membrane disruption, and recruiting and activating neutrophils. During trauma, aggregated NETs can degrade pro-inflammatory factors, which is conducive to wound repair. When NETs accumulate overly, the intracellular substances they release can trigger autoimmune reactions, cause tissue damage, intensify immune responses, and thereby give rise to diseases such as sepsis, autoimmune diseases, atherosclerosis, diabetes, and cancer. Abs, Antibodies; Agg-NETs, Aggregated NETs; AIDs, Autoimmune Diseases; AMPs, Adenosine Monophosphates; AS, Atherosclerosis; cfDNA, Circulating Free DNA; CitH3, Citrullinated histone H3; CKs, Cytokines; DM, Diabetes Mellitus; ECM, Extracellular Matrix; I/R Injury, Ischemia/reperfusion (I/R) Injury; NE, Neutrophil Elastase; pDCs, Pre-Dendritic Cells; ROS, Reactive Oxygen Species; TFs, Tissue Factors; VTE, Venous Thrombosis Embolism. (By Figdraw.).

NETs represent a critical effector mechanism of neutrophils—the body’s primary “first responders” to inflammation. When deposited in tissues, NETs establish persistent inflammatory microenvironments through sustained release of their molecular components. This chronic inflammatory signature not only reprograms the function of macrophage and dendritic cell but also drives systemic pathologies including cancer, diabetes, and atherosclerosis (3). These mechanisms establish NETs as central players in circulatory disorders and autoimmune pathogenesis (**Figure 1**). Recent paradigm shifts now recognize NETs as major contributors to chronic disease progression, mirroring the evolving role of neutrophils in cancer biology. This recognition has catalyzed targeted therapeutic strategies against NET components, demonstrating significant efficacy across infectious and inflammatory conditions.

This review focuses specifically on NETs in oncogenesis, a field significantly advanced by the landmark 2013 study of Sivan Berger-Achituv et al., which linked NETs to Ewing sarcoma (4). Although NETs retain dual potential in tumor immunity, accumulating evidence reveals their pan-cancer pro-tumorigenic functions (5). Critically, NETs drive hallmark cancer processes including cancer cell proliferation, angiogenesis, and epithelial-mesenchymal transition (EMT)—mechanisms that collectively accelerate tumor progression and adversely affect clinical prognosis. By integrating evidence on these key pro-tumorigenic mechanisms, we evaluate the emerging significance of NETs as multifunctional oncology targets.

## 2 NETosis

**Figure 2:**
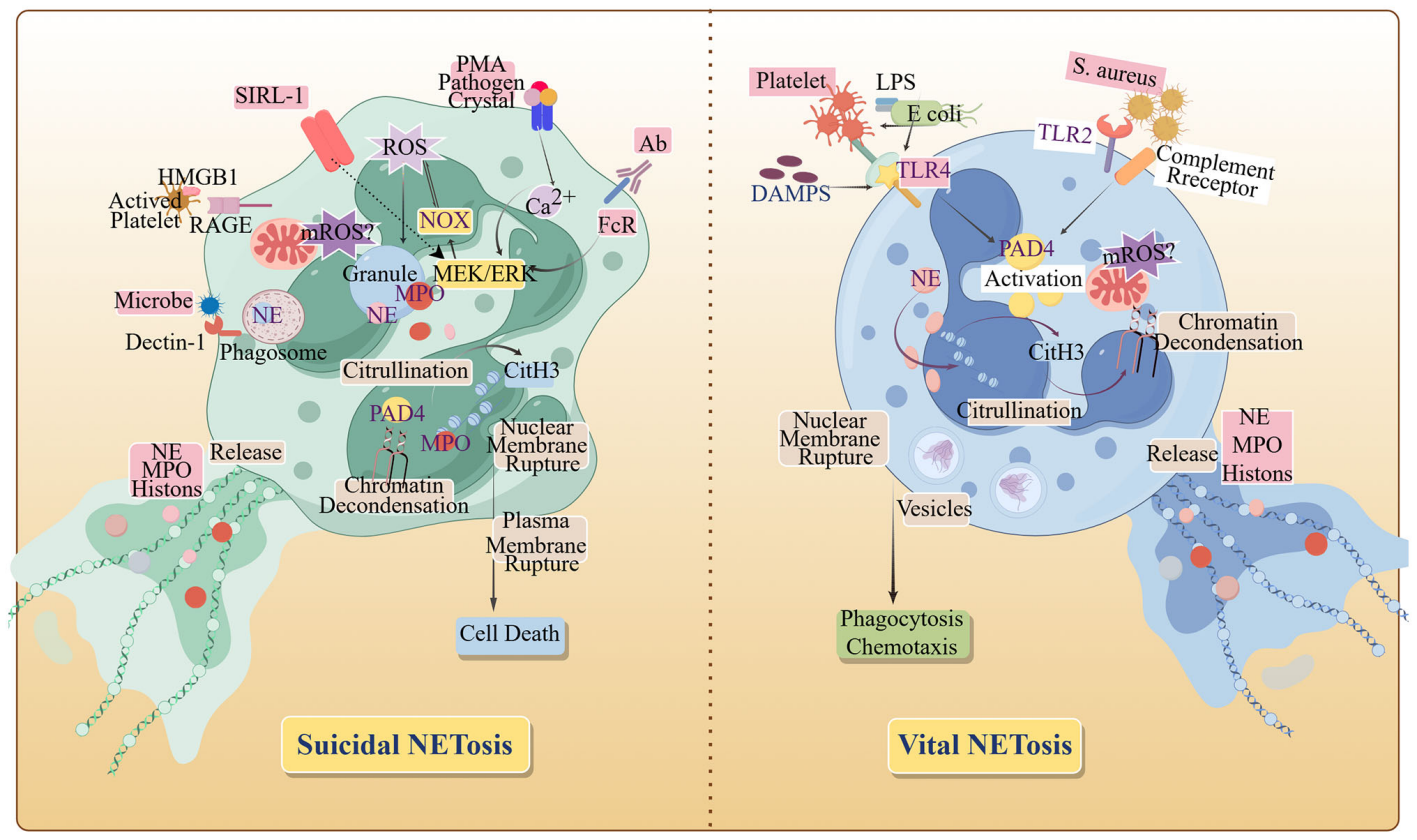
The formation pathways of NETs. When NETosis is initiated by activated neutrophils, the cytoskeleton and membrane system disintegrate, and with chromatin depolymerization, the nucleus becomes rounded, the plasma and nuclear membranes become more permeable, and granular proteins as well as chromatin and DNA are released, completing the formation of NETs. HMGB 1, High mobility group protein B 1; MPO, myeloperoxidase; NOX, NADPH Oxidase; PMA, Phorbol Myristate Acetate; PAD 4, Peptidyl Arginine Deiminase 4; RAGE, Receptor for advanced glycosylation end products; SIRL-1, Signal Inhibitory Receptor on Leukocytes-1; TLR 2, Toll-like receptor 2; TLR 4, Toll-like receptor 4. (By Figdraw.).

Neutrophil extracellular trap formation, termed NETosis (**Figure 2**), is initiated upon neutrophil activation by diverse stimuli. Originally characterized as a regulated cell death mechanism distinct from apoptosis and necrosis (6), NETosis proceeds via two established pathways: suicidal and vital NETosis. In tumors, suicidal NETosis predominates (7). This pathway involves the activation of nicotinamide adenine dinucleotide phosphate oxidase (NOX), which generate ROS that trigger peptidyl arginine deiminase 4 (PAD4)-mediated histone citrullination. Concurrently, neutrophil elastase (NE) and myeloperoxidase (MPO) translocate to the nucleus, facilitating nuclear membrane disintegration. Subsequently, chromatin decondenses, followed by the rupture of the plasma membrane, releasing the DNA-protein network and thereby causing the death of neutrophils (2). In contrast, vital NETosis involves distinct stimuli and accelerated NET release. Within 30 minutes, neutrophils extrude NETs via vesicular budding independent of NOX activity. Remarkably, these anucleated cells retain their migratory and phagocytic capacities (8).

## 3 Pro-tumorigenic functions of NETs

**Figure 3:**
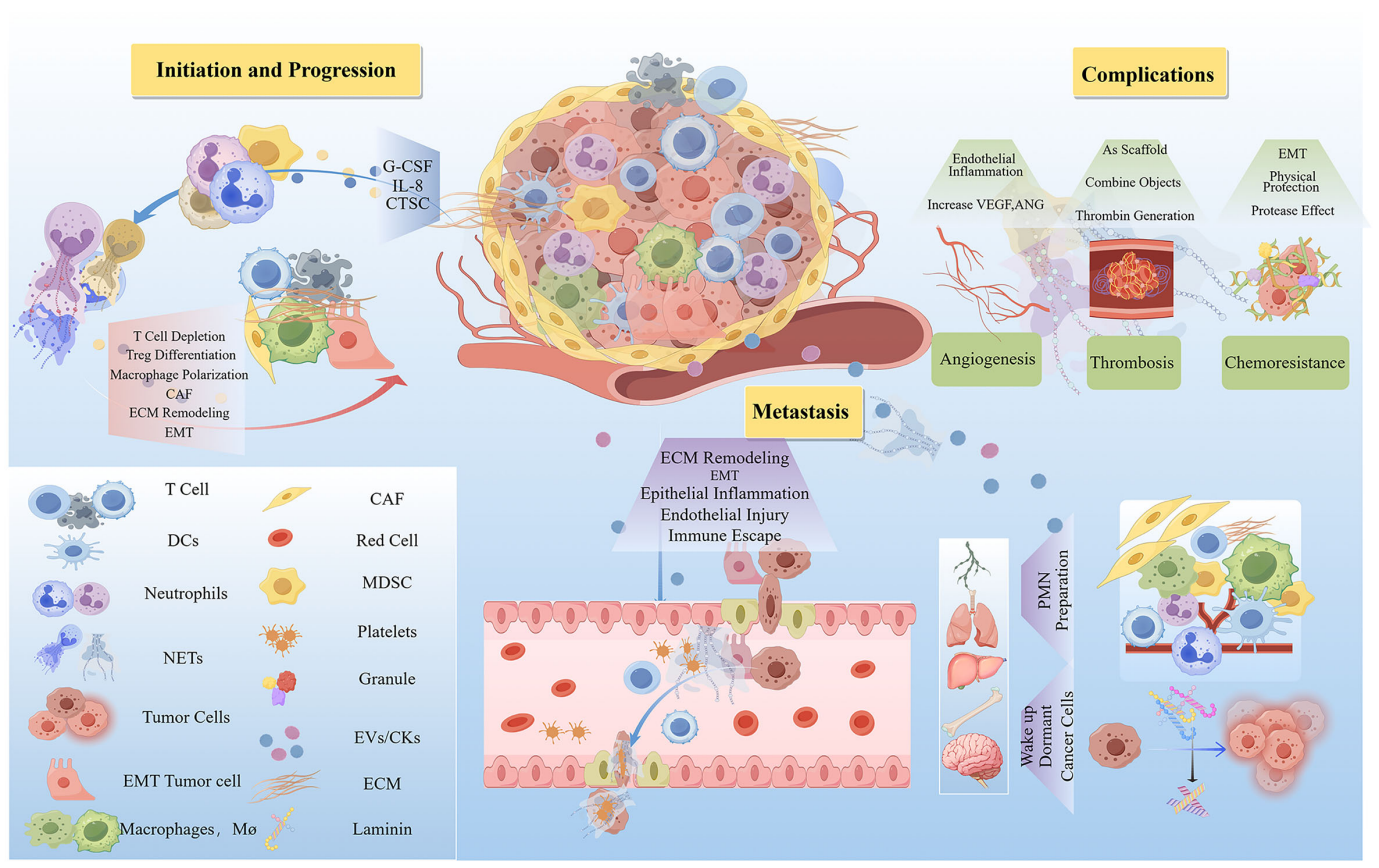
The mechanism of the carcinogenic action of NETs. NETs play a pro-cancer role by promoting cancer initiation, progression, metastasis, and complications. The quantity of NETs that promote oncogenesis is elevated in the TME. Thereby facilitating cancer initiation and progression through lymphocyte depletion, modulation of immune cell differentiation, induction of epithelial-mesenchymal transition (EMT), and remodeling of the extracellular matrix (ECM). Furthermore, NETs enhance the transvascular migration and survival of circulating tumor cells, contribute to the preparation of pre-metastatic niches (PMNs), and can reactivate dormant cancer cells to stimulate metastasis. Additionally, NETs promote angiogenesis and thrombosis, processes that are associated with drug resistance following chemotherapy. ANG, Angiopoietin; CAF, Cancer-Associated Fibroblasts; CTSC, Cathepsin C; ECM, Extracellular Matrix; EMT, Epithelial-Mesenchymal Transition; G-CSF, Granulocyte Colony-Stimulating Factor; IL-8, Interleukin 8; MDSC, Myeloid-Derived Suppressor Cells; VEGF, Vascular Endothelial Growth Factor. (By Figdraw.).

NETs exhibit dual immunological roles: they are essential for pathogen defense through microbial killing, cytokine degradation, and immune cell recruitment and regulation (9). Paradoxically, they also exert immunosuppressive effects by modulating immune cells and inflammatory mediators. These suppressive activities promote vascular occlusion, tissue damage, and disease pathogenesis. The balance between NET-mediated immune protection and immunosuppression appears to be concentration-dependent, highlighting their context-specific duality. This review focuses on the cancer-promoting immunosuppressive role of NETs (**Figure 3**).

### 3.1 NET formation induced by the tumor microenvironment

Emerging evidence reveals a complex regulatory network in which TME-derived factors orchestrate tumor-associated neutrophil (TAN) polarization and NET formation (10). The TME harbors multiple chemokines capable of inducing the release of NETs by neutrophils. One example is chitinase-3-like protein 1 (Chi3l1), a chemokine associated with triple-negative breast cancer, has been implicated in this process (11). In tumor settings, the activation of C-X-C motif chemokine receptor1 (CXCR1) and CXCR2 receptors on neutrophils by agonist C-X-C motif chemokine ligand 8/Interleukin-8(CXCL8/IL-8) stimulates neutrophil activation, thereby enhancing the formation of NET (12). Besides, tumor-produced CXCL8/IL-8 attracts human myeloid-derived suppressor cells and elicits extrusion of NETs (13). Studies have revealed that granulocyte colony-stimulating factor (G-CSF) increases the number of neutrophils prone to forming NETs in the circulation promoting tumor progression in models of chronic leukemia, breast cancer, and lung cancer (14). In thyroid cancer and melanoma, the soluble factors produced by the tumors, including but not limited to CXCL8/IL-8 and granulocyte-macrophage colony-stimulating factor (GM-CSF), can educate neutrophils to enter an activated functional state, which is related to the formation of NETs (15, 16). As well as the IL - 17-dependent recruited TAN cells subsequently form NETs in pancreatic cancer (17). Beyond chemokines, proteolytic enzymes and cytokines act synergistically: cathepsin C (CTSC) upregulates IL - 6 and CCL3 to recruit neutrophils in breast cancer while simultaneously enhancing ROS production to drive NETosis (18). Critically, these pathways form a self-amplifying cycle wherein TME factors induce NETosis, generating bioactive molecules that further stimulate neutrophil recruitment and activation. This cascade establishes a pro-tumor niche through immunosuppression, angiogenesis, and metastasis.

### 3.2 Role of NETs in tumorigenesis and progression

Chronic tissue inflammation is a well-established oncogenic driver, with NET-associated inflammation playing a significant role in tumorigenesis. In non-alcoholic steatohepatitis (NASH), elevated free fatty acids stimulate NET formation, promoting mononuclear cell infiltration and pro-inflammatory cytokine production that drive progression to hepatocellular carcinoma (HCC) (19). Similarly, infection with Fusobacterium nucleatum induces robust NET release by activated neutrophils, fostering chronic inflammation that predisposes individuals to colorectal cancer (CRC) and shapes a pro-tumorigenic TME (20).

Within the TME, NETs exert multifaceted oncogenic effects through immunomodulatory and structural remodeling. Studies across non-small cell lung cancer, bladder cancer, and metastatic melanoma demonstrate NET-mediated immunosuppression via CD8+ T cell depletion, programmed death 1 (PD - 1)- and protease-mediated T cell dysfunction (21), and physical tumor shielding through their reticular architecture. Beyond direct cytotoxic cell modulation, NETs promote broader immunosuppressive networks by inducing regulatory T (Treg) cell differentiation (22) and facilitating macrophage polarization toward an immunosuppressive phenotype (23). Furthermore, NET-associated proteases, particularly matrix metalloproteinases (MMPs), significantly remodel the extracellular matrix (ECM) through targeted protein degradation and fibrillar matrix reorganization (24, 25). This multifaceted regulation of both immune and structural components establishes NETs as central mediators of tumor-permissive niche formation across diverse malignancies.

### 3.3 NET-driven mechanisms of metastatic progression

Metastatic potential is governed by several critical factors: the invasiveness of cancer cells, their capacity to intravasate and survive the circulatory system, the establishment of pre-metastatic niches (PMNs), and the reactivation efficiency of dormant disseminated tumor cells. NETs potently enhance this metastatic cascade - the most lethal aspect of malignancy - by actively facilitating multiple steps of cancer dissemination.

NETs promote cancer cell proliferation by modulating tumor-associated inflammation, inducing EMT, and remodeling the ECM. NET-mediated inflammation upregulates cyclooxygenase-2 (COX - 2) in liver cancer cells and activates the inflammasome pathway in lung cancer cells, thereby promoting the metastatic potential of cancer cells (26). This persistent inflammatory state drives EMT, thereby transforming epithelial-like cancer cells into a mesenchymal-like phenotype with enhanced migratory and invasive capabilities (27–29). Notably, this intermediate state exhibits stem cell-like properties, which further augmenting metastatic potential. Additionally, NET-derived proteases—such as NE, MMPs, and disintegrins—degrade ECM components, releasing bioactive metabolites that contribute to a tumor-permissive microenvironment.

NETs facilitate hematogenous dissemination by promoting tumor cell migration through the vasculature. Histones within NETs directly damage vascular endothelium, creating intravasation pathways for metastatic cells (30). The binding of NET-DNA to the receptor CCDC25 enhances the mobility of circulating tumor cells (CTCs) (31), while NET-derived proteases cleave laminin, activating integrin-mediated signaling pathways that promote tumor cell survival in circulation (32). Within microvascular beds, NETs cooperate with platelets to form protective aggregates that shield micrometastases from shear stress and natural killer (NK) cell-mediated clearance (33). Despite these protective mechanisms, only a small number of CTCs manage to survive in circulation. These surviving cells continually adapt their phenotypes and may even enter a reversible dormant state to facilitate ultimate colonization (34). Ultimately, NETs enable extravasation through endothelial damage and proteolytic disruption of intercellular junctions (35).

Before the arrival of CTCs, NETs precondition future colonization sites by promoting vascular leakage, redistributing resident and bone marrow-derived cells, and remodeling the ECM—collectively establishing the PMN (36). NET components (elevated tissue factor and exposed phosphatidylserine) exert coagulant effects in cancer through enhanced thrombin and fibrin generation, promoting hypercoagulability (37). NET-associated neovascularization exhibits compromised barrier function, facilitating vascular leakage—particularly when elevated histones and fibrin damage endothelium (38). The establishment of PMNs depends on bone marrow-derived cells and frequently reprograms local stromal cells at metastatic sites. In pancreatic ductal adenocarcinoma with liver metastasis, cancer-associated fibroblasts (CAFs) originate from hepatic stellate cells (HSCs). NETs facilitate CAF recruitment by altering HSC migration (39). Subsequently, CAFs produce amyloid-β, which induces additional NET formation, establishing a feedback loop that amplifies stromal activation and promotes PMN development (40). Similarly, in breast cancer lung metastasis, NETs enhance PMN formation by provoking inflammation in alveolar epithelial cells (41). Beyond modifying resident cells, NETs shape the hepatic PMN in colorectal cancer by mediating translocation of gut microbiota and related signaling events (42).

Upon colonization of the PMN, CTCs are influenced by NETs through two primary mechanisms: NET-secreted NE and MMP - 9 degrade extracellular mucoproteins, thereby reactivating dormant cancer cells and promoting their proliferation and metastasis to distant organs (43); concurrently, NET-induced angiogenesis stimulates tumor cell division and metastatic expansion.

### 3.4 NETs in tumor-associated complications

NETs exacerbate critical cancer complications including angiogenesis, thrombosis, and therapeutic resistance, all of which contribute to increased patient mortality. NETs promote tumor angiogenesis (44) by damaging the vascular endothelium, inducing a pro-angiogenic inflammatory response (45), activating the stimulator of interferon genes (STING) pathway (46), and upregulating vascular endothelial growth factor A (VEGFA) expression (47).Within tumors, NETs exert dual pro-thrombotic functions: they combin with tissue factors to amplify thrombin generation (48, 49), and providing scaffolds for platelet and fibrin accrual via histone-mediated platelet activation and von Willebrand factor (vWF) release (14). Furthermore, NETs form physical barriers around tumor cells, shielding them from therapeutic agents and promoting the emergence of drug-resistant phenotypes (50). Simultaneously, NET-associated integrins and MMP - 9 drive EMT, which further augments chemoresistance in cancer cells (51).

## 4 NETs in cancer: diagnostic and therapeutic applications

The clinical relevance of NETs continues to expand, with their components serving as dual-purpose biomarkers for both tumor prediction and prognosis, as well as therapeutic targets. Combined strategies that incorporate NET modulation alongside immunotherapy, in conjunction with precision oncology methods, demonstrate growing therapeutic potential.

### 4.1 NET components as cancer biomarkers

Cancer transcriptome analyses identify NETs as significant oncological risk factors. NET-derived biomarkers demonstrate prognostic and therapeutic response predictive value (**Table 1**). Current detection primarily employs staining techniques, with colocalization methods preferred over single-marker assays for enhanced specificity. Enzyme-linked immunosorbent assay (ELISA) quantifies MPO-DNA complexes and citrullinated fibrinogen in biofluids (52), while multiplexed ELISA/immunofluorescence platforms measure plasma NET components using MPO, citrullinated histone H3 (CitH3), and DNA antibodies (53). Flow cytometry with SYTOX Green/anti-MPO double-staining provides superior objectivity for *in vivo* and *in vitro* NET detection. Emerging techniques include NET transcriptomic signatures and computed tomography (CT)-derived radiomic models, which have been validated for predicting HCC prognosis and immunotherapy responses (54).

**Table 1:** Markers used to detect NETs.

Detection object	Source	References
NE、MPO	Granin	(5, 89)
S100A8、S100A9	Intracellular calcaretin of neutrophils	(90)
PAD4	Protease in the nucleus	(91)
CitH3	Citrullinated Histone	(92)
The cleavage site of CitH3-3D9	The specific cleavage site of NETs	(93)
cf-DNA	Depolymerized chromatin	(31)
TRIM8	Genes associated with childhood acute lymphoblastic leukemia	(94)

Despite rapid methodological advances, the detection of NETs remains constrained by several critical limitations. The lack of specificity in circulating free DNA (cfDNA) poses a major challenge, as it originates from both NETs and non-NET cellular debris. Furthermore, conventional microscopic evaluations are susceptible to observer bias, while currently available antibody panels often fail to reliably detect NETs even when multiplexed approaches are employed. Additionally, the dynamic and heterogeneous kinetics of NETosis further reduce detection accuracy (55). Ito address these challenges, newly developed automated detection systems have emerged, offering enhanced reproducibility and enabling more profound mechanistic insights into NET-related pathophysiology.

### 4.2 Therapeutic targeting of NETs in oncology

The growing recognition of NET pathophysiology has spurred clinical exploration of NET-targeted therapies, which leverage anti-inflammatory mechanisms, immune modulation, thrombo-regulation, and ROS control. These strategies aim to suppress NETosis, thereby mitigating NET-mediated pathology and improving patient survival. Recent oncology research highlights critical NET-cancer interplay. Since systemic neutrophil depletion carries a risk of life-threatening neutropenia, strategies that target NET structures post-neutrophil activation circumvent this limitation and represent a promising immunotherapeutic direction. Specifically, the degradation of key NET components and the inhibition of NET formation offer rational and targeted approaches for cancer therapy, as summarized in **Table 2**.

**Table 2:** Methods and applications of targeted NETs to treat cancer.

Target		Mechanism	Application	References
Enzymes	PAD4	Inhibitor	Cl-amidine and F-amidine GSK484 JBI-589 Multifunctional nanoagent	(95) (96) (56) (57)
	NOX	Inhibitor	Taurine GSK2795039 DPI	(63) (64) (65)
	NE	Inhibitor	Flavonoids Tetrahydropyrimidine derivatives Compounds containing boron or sulfur and fluorine bonds Inhalable NE Inhibitors Engineered Exocrine	(58) (59) (60) (61) (62)
DNA Cleavage	DNase	Endonuclease	DNase I DNase I MG Dual pH responsive hydrogels Bionic nanocarrie Injectable combination hydrogel Recombinant human DNase I	(66) (67) (68) (69) (70) (71)
Formation of NETs	TLR 4	Inhibitor	TAK-242	(75)
	CXCR	Antagonist	AMD3100	(74)
	ROS	Blocker	Curcumin	(79)
	HMGB 1	Inhibitor	Glycyrrhizin Metformin	(76) (77)
	TLR 9	Blocker	Hydroxychloroquine	(80)
	NETosis	Inhibitor	Glycyrrhizin Tetrahydroisoquinolines	(81) (82)
	IL-17	Antibody	BAY11-7082	(83)
	G-CSFR	Antibody	CSL324	(84)
	CCDC 25	Antibody Oncolytic bacteria	Aniti-CCDC25 VNP-shCCDC25	(49) (85)
Release of NETs	GSDMD	Inhibitor	Disulfiram	(88)

#### 4.2.1 Targeting proteolytic enzymes in cancer therapy

Inhibiting key NETosis enzymes effectively suppresses NET formation. PAD4—which catalyzes the conversion of arginine to citrulline—represents a well-validated therapeutic target. However, its irreversible inhibitors (Cl-amidine, F-amidine) and reversible counterparts (GSK484, JBI - 589) exhibit limited specificity in murine and human models (56). PAD4 inhibitor-based combination therapies show promising anticancer activity: for instance, multifunctional nano-agents integrating PAD4 inhibition with sonodynamic and immunotherapies demonstrate significant anti-metastatic efficacy (57).

Following neutrophil activation, NE is extruded extracellularly within NETs, serving as both a key NET formation mediator and effector. NE inhibitors have longstanding anticancer applications: naturally derived flavonoids and synthetic tetrahydropyridine derivatives demonstrate promising antitumor efficacy in experimental models (58, 59). Boron-, sulfur-, and fluorine-containing compounds—structurally analogous to heparan sulfate proteoglycan metabolites and aminoglycosides—inhibit NE activity and suppress NETosis (60). Clinically, sivelestat sodium treats acute respiratory distress syndrome, while next-generation NE inhibitors (lonodelestat/POL6014, alvelestat/MPH966) show safety in Phase I trials (49, 61). Engineered exosomal NE inhibitors further enhance antitumor immunity as *in situ* dendritic cell vaccines in breast cancer (62).

As tumor-induced NETosis is NOX-dependent, inhibiting NOX effectively suppresses NET formation. The non-selective NOX inhibitor diphenyleneiodonium chloride (DPI) and taurine attenuate ROS-dependent NETosis in murine models, delaying tumor progression (63, 64, 65).

#### 4.2.2 DNase-based therapeutic strategies

Deoxyribonuclease I (DNase I) cleaves extracellular NET-DNA, thereby inhibiting NET-driven tumor progression (66). Although the clinical application of free DNase I is constrained by its serum instability, microgel formulations incorporating hydrophilic zwitterionic modifications significantly improve its pharmacokinetic profile and bioavailability (67). The immunosuppressive effects of NETs and the acidic TME diminish the efficacy of NK cell-based therapies; however, this limitation can be overcome using pH-responsive hydrogels that co-deliver DNase I and acidity-neutralizing nanoparticles, thereby preventing HCC recurrence after resection (68). To disrupt the pro-metastatic signaling axis mediated by NET-DNA and CCDC25, lipid-based nanocarriers engineered to express CCDC25 and encapsulate DNase I have been developed, effectively suppressing colorectal cancer metastasis to the liver (69). Injectable fibrin-alginate hydrogels enabling the dual release of DNase I and propranolol simultaneously facilitate NET degradation and inhibit β-adrenergic signaling, leading to a significant reduction in postoperative recurrence and metastatic spread (70). Recombinant human DNase I—which is clinically approved for the treatment of cystic fibrosis—also exhibits therapeutic potential in mitigating cancer-associated thrombosis (71). Studies have indicated that the efficacy of NET inhibition by DNase I is highly dependent on both the dosage and the administration route (72). Moreover, preclinical studies of DNase formulations have detected corresponding antibodies in both animal models and patient sera following treatment, suggesting a potential risk of allergic reactions (73).

#### 4.2.3 Inhibition of NET formation and release

Inhibiting the formation and release of NETs effectively attenuates their pathological effects. Platelet-neutrophil interactions can trigger NETosis, which can be suppressed by blocking TLR4 expressed on platelets or CXCR2 on neutrophils (74). For instance, administration of the TLR4 inhibitor TAK - 242 significantly inhibits the proliferation of ovarian cancer cells (75). The inflammatory mediator high mobility group box 1 (HMGB1) is implicated in NETosis induction, and its inhibitors, such as glycyrrhizin and metformin, exhibit anti-inflammatory and immunomodulatory effects in chronic inflammatory diseases (76, 77). Targeting HMGB1 in gastric cancer effectively reduces NET formation and inhibits tumor growth (78). Additionally, NETosis is suppressed by several natural and synthetic compounds, including curcumin, glycyrrhizin, hydroxychloroquine, and tetrahydroisoquinolines (79, 80, 81, 82).

Within the TME, anticancer effects can be achieved by reducing neutrophil activation and interrupting NET-receptor interactions. Thus, IL - 17 and G-CSF, which recruit neutrophils and trigger NETosis, represent potential therapeutic targets. IL - 17 antibodies are already clinically used in psoriasis, and their efficacy in oncology has been preliminarily validated (83). Blocking G-CSF signaling via an anti-G-CSFR antibody (CSL324) reduces NET formation without impairing neutrophil phagocytosis or oxidative burst capacity, highlighting its potential as an immunotherapeutic target (84). Similarly, targeting the CCDC25 receptor on cancer cells disrupts its interaction with NET-DNA and inhibits metastatic progression (85).

Gasdermin D (GSDMD), which facilitates NET release, can be inhibited by disulfiram—an FDA-approved drug for chronic alcoholism (86–88). Disulfiram also significantly suppresses tumor growth by attenuating NET release, and nanoformulations based on this compound have shown promising results in experimental studies.

## 5 Conclusion

NETs, which represent an activated state of neutrophils, participate in a wide range of physiological and pathological processes. Initially recognized for their role in trapping pathogens, NETs have more recently been implicated in promoting disease. Studies indicate that a balanced rate of NET formation and clearance contributes to host defense against infection and tissue damage, whereas excessive or persistent NET formation drives pathology—including cancer. The role of NETs in cancer is subject to ongoing debate, as they exhibit both anti-tumor and pro-tumor functions in different contexts. The probable mechanisms of the anti-tumor effect are related to its direct killing of cancer cells or stimulation of the immune system to fight against the tumor. Under the bidirectional interplay between malignant tumors and immune cells, the effects caused by the quantity and composition of NETs change. With the weakening of immune surveillance, NETs exhibit pro-tumor effect and actively modulate oncogenic progression through multifaceted roles. Owing to these pro-tumorigenic roles, NET-targeted therapeutic strategies are gaining attention in cancer immunotherapy. NETs are increasingly regarded both as predictive biomarkers and therapeutic targets in oncology.

However, when discussing NET-targeted therapies, the potential risks of immunosuppression and increased infection must be carefully considered. Furthermore, imprecise tumor classification remains an obstacle to the development and application of targeted therapies. The inherent difficulty in clearly detecting NET remains a key limitation that hinders progress in evaluating the effectiveness of related pharmacological interventions. Current priorities include the clinical translation of NET-directed agents, with DNase I, PAD4 inhibitors, and disulfiram representing leading candidates. Although progress remains limited and numerous challenges persist, ongoing research is essential to elucidate the tumor-specific functions of NETs. Deeper insight into their systemic and local effects within the TME will support the development of selective interventions that suppress protumor activities without impairing antitumor immunity, thereby contributing to more effective immunotherapeutic approaches.

## References

[1] Anker P Stroun M Maurice PA. Spontaneous release of DNA by human blood lymphocytes as shown in an *in vitro* system. Cancer Res. (1975) 35:2375–82., PMID: 1149042

[2] Brinkmann V Reichard U Goosmann C Fauler B Uhlemann Y Weiss DS. Neutrophil extracellular traps kill bacteria. Sci (New York NY). (2004) 303:1532–5. doi: 10.1126/science.1092385, PMID: 15001782

[3] Papayannopoulos V. Neutrophil extracellular traps in immunity and disease. Nat Rev Immunol. (2018) 18:134–47. doi: 10.1038/nri.2017.105, PMID: 28990587

[4] Berger-Achituv S Brinkmann V Abed UA Kühn LI Ben-Ezra J Elhasid R. A proposed role for neutrophil extracellular traps in cancer immunoediting. Front Immunol. (2013) 4:48. doi: 10.3389/fimmu.2013.00048, PMID: 23508552 PMC3589747

[5] Zhang Y Guo L Dai Q Shang B Xiao T Di X. A signature for pan-cancer prognosis based on neutrophil extracellular traps. J immunotherapy Cancer. (2022) 10:e004210. doi: 10.1136/jitc-2021-004210, PMID: 35688556 PMC9189842

[6] Adrover JM McDowell SAC He XY Quail DF Egeblad M. NETworking with cancer: The bidirectional interplay between cancer and neutrophil extracellular traps. Cancer Cell. (2023) 41:505–26. doi: 10.1016/j.ccell.2023.02.001, PMID: 36827980 PMC10280682

[7] Demkow U. Neutrophil extracellular traps (NETs) in cancer invasion, evasion and metastasis. Cancers. (2021) 13:4495. doi: 10.3390/cancers13174495, PMID: 34503307 PMC8431228

[8] Wang Y Du C Zhang Y Zhu L. Composition and function of neutrophil extracellular traps. Biomolecules. (2024) 14:416. doi: 10.3390/biom14040416, PMID: 38672433 PMC11048602

[9] Monteith AJ Miller JM Maxwell CN Chazin WJ Skaar EP. Neutrophil extracellular traps enhance macrophage killing of bacterial pathogens. Sci Adv. (2021) 7:eabj2101. doi: 10.1126/sciadv.abj2101, PMID: 34516771 PMC8442908

[10] Fang Q Stehr AM Naschberger E Knopf J Herrmann M Stürzl M. No NETs no TIME: Crosstalk between neutrophil extracellular traps and the tumor immune microenvironment. Front Immunol. (2022) 13:1075260. doi: 10.3389/fimmu.2022.1075260, PMID: 36618417 PMC9816414

[11] Taifour T Attalla SS Zuo D Gu Y Sanguin-Gendreau V Proud H. The tumor-derived cytokine Chi3l1 induces neutrophil extracellular traps that promote T cell exclusion in triple-negative breast cancer. Immunity. (2023) 56:2755–72.e8. doi: 10.1016/j.immuni.2023.11.002, PMID: 38039967

[12] Teijeira Á Garasa S Gato M Alfaro C Migueliz I Cirella A. CXCR1 and CXCR2 chemokine receptor agonists produced by tumors induce neutrophil extracellular traps that interfere with immune cytotoxicity. Immunity. (2020) 52:856–71.e8. doi: 10.1016/j.immuni.2020.03.001, PMID: 32289253

[13] Alfaro C Teijeira A Oñate C Pérez G Sanmamed MF Andueza MP. Tumor-produced interleukin-8 attracts human myeloid-derived suppressor cells and elicits extrusion of neutrophil extracellular traps (NETs). Clin Cancer research: an Off J Am Assoc Cancer Res. (2016) 22:3924–36. doi: 10.1158/1078-0432.CCR-15-2463, PMID: 26957562

[14] Demers M Krause DS Schatzberg D Martinod K Voorhees JR Fuchs TA. Cancers predispose neutrophils to release extracellular DNA traps that contribute to cancer-associated thrombosis. Proc Natl Acad Sci United States America. (2012) 109:13076–81. doi: 10.1073/pnas.1200419109, PMID: 22826226 PMC3420209

[15] Galdiero MR Varricchi G Loffredo S Bellevicine C Lansione T Ferrara AL. Potential involvement of neutrophils in human thyroid cancer. PloS One. (2018) 13:e0199740. doi: 10.1371/journal.pone.0199740, PMID: 29953504 PMC6023126

[16] Modestino L Cristinziano L Trocchia M Ventrici A Capone M Madonna G. Melanoma-derived soluble mediators modulate neutrophil biological properties and the release of neutrophil extracellular traps. Cancer immunology immunotherapy: CII. (2023) 72:3363–76. doi: 10.1007/s00262-023-03493-5, PMID: 37525065 PMC10491523

[17] Zhang Y Chandra V Riquelme Sanchez E Dutta P Quesada PR Rakoski A. Interleukin-17-induced neutrophil extracellular traps mediate resistance to checkpoint blockade in pancreatic cancer. J Exp Med. (2020) 217(12):e20190354. doi: 10.1084/jem.20190354, PMID: 32860704 PMC7953739

[18] Xiao Y Cong M Li J He D Wu Q Tian P. Cathepsin C promotes breast cancer lung metastasis by modulating neutrophil infiltration and neutrophil extracellular trap formation. Cancer Cell. (2021) 39:423–37.e7. doi: 10.1016/j.ccell.2020.12.012, PMID: 33450198

[19] van der Windt DJ Sud V Zhang H Varley PR Goswami J Yazdani HO. Neutrophil extracellular traps promote inflammation and development of hepatocellular carcinoma in nonalcoholic steatohepatitis. Hepatol (Baltimore Md). (2018) 68:1347–60. doi: 10.1002/hep.29914, PMID: 29631332 PMC6173613

[20] Kong X Zhang Y Xiang L You Y Duan Y Zhao Y. Fusobacterium nucleatum-triggered neutrophil extracellular traps facilitate colorectal carcinoma progression. J Exp Clin Cancer research: CR. (2023) 42:236. doi: 10.1186/s13046-023-02817-8, PMID: 37684625 PMC10492297

[21] Kaltenmeier C Yazdani HO Morder K Geller DA Simmons RL Tohme S. Neutrophil extracellular traps promote T cell exhaustion in the tumor microenvironment. Front Immunol. (2021) 12:785222. doi: 10.3389/fimmu.2021.785222, PMID: 34899751 PMC8652262

[22] Wang H Zhang H Wang Y Brown ZJ Xia Y Huang Z. Regulatory T-cell and neutrophil extracellular trap interaction contributes to carcinogenesis in non-alcoholic steatohepatitis. J hepatology. (2021) 75:1271–83. doi: 10.1016/j.jhep.2021.07.032, PMID: 34363921 PMC12888775

[23] Yu C Zhou G Shi Z Yu L Zhou X. TREM1 facilitates the development of gastric cancer through regulating neutrophil extracellular traps-mediated macrophage polarization. Digestive liver disease: Off J Ital Soc Gastroenterol Ital Assoc Study Liver. (2024) 56:1237–47. doi: 10.1016/j.dld.2023.12.002, PMID: 38151453

[24] Dongre A Weinberg RA. New insights into the mechanisms of epithelial-mesenchymal transition and implications for cancer. Nat Rev Mol Cell Biol. (2019) 20:69–84. doi: 10.1038/s41580-018-0080-4, PMID: 30459476

[25] Miller-Ocuin JL Liang X Boone BA Doerfler WR Singhi AD Tang D. DNA released from neutrophil extracellular traps (NETs) activates pancreatic stellate cells and enhances pancreatic tumor growth. Oncoimmunology. (2019) 8:e1605822. doi: 10.1080/2162402X.2019.1605822, PMID: 31428515 PMC6685506

[26] Wang Y Liu F Chen L Fang C Li S Yuan S. Neutrophil extracellular traps (NETs) promote non-small cell lung cancer metastasis by suppressing lncRNA MIR503HG to activate the NF-κB/NLRP3 inflammasome pathway. Front Immunol. (2022) 13:867516. doi: 10.3389/fimmu.2022.867516, PMID: 35707534 PMC9190762

[27] Martins-Cardoso K Almeida VH Bagri KM Rossi MID Mermelstein CS König S. Neutrophil extracellular traps (NETs) promote pro-metastatic phenotype in human breast cancer cells through epithelial-mesenchymal transition. Cancers. (2020) 12(6):1542. doi: 10.3390/cancers12061542, PMID: 32545405 PMC7352979

[28] Pieterse E Rother N Garsen M Hofstra JM Satchell SC Hoffmann M. Neutrophil extracellular traps drive endothelial-to-mesenchymal transition. Arteriosclerosis thrombosis Vasc Biol. (2017) 37:1371–9. doi: 10.1161/ATVBAHA.117.309002, PMID: 28495931

[29] Zhu T Zou X Yang C Li L Wang B Li R. Neutrophil extracellular traps promote gastric cancer metastasis by inducing epithelial−mesenchymal transition. Int J Mol Med. (2021) 48(1):127. doi: 10.3892/ijmm.2021.4960, PMID: 34013374 PMC8128417

[30] Saffarzadeh M Juenemann C Queisser MA Lochnit G Barreto G Galuska SP. Neutrophil extracellular traps directly induce epithelial and endothelial cell death: a predominant role of histones. PloS One. (2012) 7:e32366. doi: 10.1371/journal.pone.0032366, PMID: 22389696 PMC3289648

[31] Yang L Liu Q Zhang X Liu X Zhou B Chen J. DNA of neutrophil extracellular traps promotes cancer metastasis via CCDC25. Nature. (2020) 583:133–8. doi: 10.1038/s41586-020-2394-6, PMID: 32528174

[32] Najmeh S Cools-Lartigue J Rayes RF Gowing S Vourtzoumis P Bourdeau F. Neutrophil extracellular traps sequester circulating tumor cells via β1-integrin mediated interactions. Int J cancer. (2017) 140:2321–30. doi: 10.1002/ijc.30635, PMID: 28177522

[33] Ren J He J Zhang H Xia Y Hu Z Loughran P. Platelet TLR4-ERK5 axis facilitates NET-mediated capturing of circulating tumor cells and distant metastasis after surgical stress. Cancer Res. (2021) 81:2373–85. doi: 10.1158/0008-5472.CAN-20-3222, PMID: 33687949 PMC8137664

[34] Gerstberger S Jiang Q Ganesh K. Metastasis. Cell. (2023) 186:1564–79. doi: 10.1016/j.cell.2023.03.003, PMID: 37059065 PMC10511214

[35] Ma Y Yang X Chatterjee V Meegan JE Beard RS Jr. Yuan SY. Role of neutrophil extracellular traps and vesicles in regulating vascular endothelial permeability. Front Immunol. (2019) 10:1037. doi: 10.3389/fimmu.2019.01037, PMID: 31143182 PMC6520655

[36] Peinado H Zhang H Matei IR Costa-Silva B Hoshino A Rodrigues G. Pre-metastatic niches: organ-specific homes for metastases. Nat Rev Cancer. (2017) 17:302–17. doi: 10.1038/nrc.2017.6, PMID: 28303905

[37] Jin J Wang F Tian J Zhao X Dong J Wang N. Neutrophil extracellular traps contribute to coagulopathy after traumatic brain injury. JCI Insight. (2023) 8(6):e141110. doi: 10.1172/jci.insight.141110, PMID: 36802340 PMC10070118

[38] Poto R Cristinziano L Modestino L de Paulis A Marone G Loffredo S. Neutrophil extracellular traps, angiogenesis and cancer. Biomedicines. (2022) 10(2):431. doi: 10.3390/biomedicines10020431, PMID: 35203640 PMC8962440

[39] Takesue S Ohuchida K Shinkawa T Otsubo Y Matsumoto S Sagara A. Neutrophil extracellular traps promote liver micrometastasis in pancreatic ductal adenocarcinoma via the activation of cancer−associated fibroblasts. Int J Oncol. (2020) 56:596–605. doi: 10.3892/ijo.2019.4951, PMID: 31894273

[40] Munir H Jones JO Janowitz T Hoffmann M Euler M Martins CP. Stromal-driven and Amyloid β-dependent induction of neutrophil extracellular traps modulates tumor growth. Nat Commun. (2021) 12:683. doi: 10.1038/s41467-021-20982-2, PMID: 33514748 PMC7846803

[41] Zeng Z Xu S Wang F Peng X Zhang W Zhan Y. HAO1-mediated oxalate metabolism promotes lung pre-metastatic niche formation by inducing neutrophil extracellular traps. Oncogene. (2022) 41:3719–31. doi: 10.1038/s41388-022-02248-3, PMID: 35739335 PMC9287177

[42] Wu J Dong W Pan Y Wang J Wu M Yu Y. Crosstalk between gut microbiota and metastasis in colorectal cancer: implication of neutrophil extracellular traps. Front Immunol. (2023) 14:1296783. doi: 10.3389/fimmu.2023.1296783, PMID: 37936694 PMC10626548

[43] Albrengues J Shields MA Ng D Park CG Ambrico A Poindexter ME. Neutrophil extracellular traps produced during inflammation awaken dormant cancer cells in mice. Sci (New York NY). (2018) 361(6409):eaao4227. doi: 10.1126/science.aao4227, PMID: 30262472 PMC6777850

[44] Yang S Sun B Li J Li N Zhang A Zhang X. Neutrophil extracellular traps promote angiogenesis in gastric cancer. Cell communication signaling: CCS. (2023) 21:176. doi: 10.1186/s12964-023-01196-z, PMID: 37480055 PMC10362668

[45] Aldabbous L Abdul-Salam V McKinnon T Duluc L Pepke-Zaba J Southwood M. Neutrophil extracellular traps promote angiogenesis: evidence from vascular pathology in pulmonary hypertension. Arteriosclerosis thrombosis Vasc Biol. (2016) 36:2078–87. doi: 10.1161/ATVBAHA.116.307634, PMID: 27470511

[46] Kang L Yu H Yang X Zhu Y Bai X Wang R. Neutrophil extracellular traps released by neutrophils impair revascularization and vascular remodeling after stroke. Nat Commun. (2020) 11:2488. doi: 10.1038/s41467-020-16191-y, PMID: 32427863 PMC7237502

[47] Zhang C Wu D Dong B Liao G Yu Y Huang S. The scaffold of neutrophil extracellular traps promotes CCA progression and modulates angiogenesis via ITGAV/NFκB. Cell communication signaling: CCS. (2024) 22:103. doi: 10.1186/s12964-024-01500-5, PMID: 38326837 PMC10851487

[48] Herre M Cedervall J Mackman N Olsson AK. Neutrophil extracellular traps in the pathology of cancer and other inflammatory diseases. Physiol Rev. (2023) 103:277–312. doi: 10.1152/physrev.00062.2021, PMID: 35951483 PMC9576172

[49] Cristinziano L Modestino L Antonelli A Marone G Simon HU Varricchi G. Neutrophil extracellular traps in cancer. Semin Cancer Biol. (2022) 79:91–104. doi: 10.1016/j.semcancer.2021.07.011, PMID: 34280576

[50] Shahzad MH Feng L Su X Brassard A Dhoparee-Doomah I Ferri LE. Neutrophil extracellular traps in cancer therapy resistance. Cancers. (2022) 14. doi: 10.3390/cancers14051359, PMID: 35267667 PMC8909607

[51] Mousset A Lecorgne E Bourget I Lopez P Jenovai K Cherfils-Vicini J. Neutrophil extracellular traps formed during chemotherapy confer treatment resistance via TGF-β activation. Cancer Cell. (2023) 41:757–75.e10. doi: 10.1016/j.ccell.2023.03.008, PMID: 37037615 PMC10228050

[52] Sue T Ichikawa T Hattori S Otani H Fujimura S Higuchi T. Quantitative evaluation of citrullinated fibrinogen for detection of neutrophil extracellular traps. Immunologic Res. (2024) 72:409–17. doi: 10.1007/s12026-023-09446-5, PMID: 38087184

[53] Matta B Battaglia J Barnes BJ. Detection of neutrophil extracellular traps in patient plasma: method development and validation in systemic lupus erythematosus and healthy donors that carry IRF5 genetic risk. Front Immunol. (2022) 13:951254. doi: 10.3389/fimmu.2022.951254, PMID: 35958624 PMC9360330

[54] Xin H Lai Q Zhou Y He J Song Y Liao M. Noninvasive evaluation of neutrophil extracellular traps signature predicts clinical outcomes and immunotherapy response in hepatocellular carcinoma. Front Immunol. (2023) 14:1134521. doi: 10.3389/fimmu.2023.1134521, PMID: 37520528 PMC10374215

[55] Tan C Aziz M Wang P. The vitals of NETs. J Leukoc Biol. (2021) 110:797–808. doi: 10.1002/JLB.3RU0620-375R, PMID: 33378572 PMC9059135

[56] Deng H Lin C Garcia-Gerique L Fu S Cruz Z Bonner EE. A novel selective inhibitor JBI - 589 targets PAD4-mediated neutrophil migration to suppress tumor progression. Cancer Res. (2022) 82:3561–72. doi: 10.1158/0008-5472.CAN-21-4045, PMID: 36069973 PMC9532374

[57] Zhu D Lu Y Yang S Hu T Tan C Liang R. PAD4 inhibitor-functionalized layered double hydroxide nanosheets for synergistic sonodynamic therapy/immunotherapy of tumor metastasis. Advanced Sci (Weinheim Baden-Wurttemberg Germany). (2024) 11:e2401064. doi: 10.1002/advs.202401064, PMID: 38708711 PMC11234469

[58] Jakimiuk K Gesek J Atanasov AG Tomczyk M. Flavonoids as inhibitors of human neutrophil elastase. J Enzyme inhibition medicinal Chem. (2021) 36:1016–28. doi: 10.1080/14756366.2021.1927006, PMID: 33980119 PMC8128182

[59] Patel A Gandhi K Shah S Patel D Chhatbar S Shah D. In silico Study and Solvent-free one-pot Synthesis of Tetrahydropyrimidine derivatives by Mechanochemistry Approach for Targeting Human Neutrophil Elastase against Lung Cancer. Curr computer-aided Drug design. (2022) 18:293–306. doi: 10.2174/1573409918666220622232501, PMID: 35747983

[60] Donarska B Z Łączkowski K. Recent advances in the development of elastase inhibitors. Future medicinal Chem. (2020) 12:1809–13. doi: 10.4155/fmc-2020-0163, PMID: 33016117

[61] Barth P Bruijnzeel P Wach A Sellier Kessler O Hooftman L Zimmermann J. Single dose escalation studies with inhaled POL6014, a potent novel selective reversible inhibitor of human neutrophil elastase, in healthy volunteers and subjects with cystic fibrosis. J cystic fibrosis: Off J Eur Cystic Fibrosis Society. (2020) 19:299–304. doi: 10.1016/j.jcf.2019.08.020, PMID: 31501052

[62] Huang L Rong Y Tang X Yi K Qi P Hou J. Engineered exosomes as an in *situ* DC-primed vaccine to boost antitumor immunity in breast cancer. Mol cancer. (2022) 21:45. doi: 10.1186/s12943-022-01515-x, PMID: 35148751 PMC8831689

[63] Li M Gao Y Wang Z Wu B Zhang J Xu Y. Taurine inhibits Streptococcus uberis-induced NADPH oxidase-dependent neutrophil extracellular traps via TAK1/MAPK signaling pathways. Front Immunol. (2022) 13:927215. doi: 10.3389/fimmu.2022.927215, PMID: 36148229 PMC9488113

[64] Leung HHL Perdomo J Ahmadi Z Yan F McKenzie SE Chong BH. Inhibition of NADPH oxidase blocks NETosis and reduces thrombosis in heparin-induced thrombocytopenia. Blood advances. (2021) 5:5439–51. doi: 10.1182/bloodadvances.2020003093, PMID: 34478504 PMC9153028

[65] Zhu W Yang S Meng D Wang Q Ji J. Targeting NADPH oxidase and integrin α5β1 to inhibit neutrophil extracellular traps-mediated metastasis in colorectal cancer. Int J Mol Sci. (2023) 24(21):16001. doi: 10.3390/ijms242116001, PMID: 37958984 PMC10650826

[66] Park J Wysocki RW Amoozgar Z Maiorino L Fein MR Jorns J. Cancer cells induce metastasis-supporting neutrophil extracellular DNA traps. Sci Trans Med. (2016) 8:361ra138. doi: 10.1126/scitranslmed.aag1711, PMID: 27798263 PMC5550900

[67] Hosseinnejad A Ludwig N Wienkamp AK Rimal R Bleilevens C Rossaint R. DNase I functional microgels for neutrophil extracellular trap disruption. Biomaterials science. (2021) 10:85–99. doi: 10.1039/D1BM01591E, PMID: 34812809

[68] Cheng Y Gong Y Chen X Zhang Q Zhang X He Y. Injectable adhesive hemostatic gel with tumor acidity neutralizer and neutrophil extracellular traps lyase for enhancing adoptive NK cell therapy prevents post-resection recurrence of hepatocellular carcinoma. Biomaterials. (2022) 284:121506. doi: 10.1016/j.biomaterials.2022.121506, PMID: 35390709

[69] Wang Z Chen C Shi C Zhao X Gao L Guo F. Cell membrane derived liposomes loaded with DNase I target neutrophil extracellular traps which inhibits colorectal cancer liver metastases. J Controlled release: Off J Controlled Release Society. (2023) 357:620–9. doi: 10.1016/j.jconrel.2023.04.013, PMID: 37061194

[70] Zhou H Zhu C Zhao Q Ni J Zhang H Yang G. Wrecking neutrophil extracellular traps and antagonizing cancer-associated neurotransmitters by interpenetrating network hydrogels prevent postsurgical cancer relapse and metastases. Bioactive materials. (2024) 39:14–24. doi: 10.1016/j.bioactmat.2024.05.022, PMID: 38783926 PMC11112132

[71] Várady CBS Oliveira AC Monteiro RQ Gomes T. Recombinant human DNase I for the treatment of cancer-associated thrombosis: A pre-clinical study. Thromb Res. (2021) 203:131–7. doi: 10.1016/j.thromres.2021.04.028, PMID: 34015562

[72] Wang CL Wang Y Jiang QL Zeng Y Yao QP Liu X. DNase I and sivelestat ameliorate experimental hindlimb ischemia-reperfusion injury by eliminating neutrophil extracellular traps. J Inflammation Res. (2023) 16:707–21. doi: 10.2147/JIR.S396049, PMID: 36852300 PMC9961174

[73] Wolff RK Blanchard JD. Preclinical safety. J Aerosol Med Pulmonary Drug Delivery. (2025) 38:136–44. doi: 10.1089/jamp.2025.52511.isam, PMID: 40445826

[74] Ngamsri KC Putri RA Jans C Schindler K Fuhr A Zhang Y. CXCR4 and CXCR7 inhibition ameliorates the formation of platelet-neutrophil complexes and neutrophil extracellular traps through adora2b signaling. Int J Mol Sci. (2021) 22(24):13576. doi: 10.3390/ijms222413576, PMID: 34948374 PMC8709064

[75] Kashani B Zandi Z Bashash D Zaghal A Momeny M Poursani EM. Small molecule inhibitor of TLR4 inhibits ovarian cancer cell proliferation: new insight into the anticancer effect of TAK - 242 (Resatorvid). Cancer chemotherapy Pharmacol. (2020) 85:47–59. doi: 10.1007/s00280-019-03988-y, PMID: 31786654

[76] Gu J Ran X Deng J Zhang A Peng G Du J. Glycyrrhizin alleviates sepsis-induced acute respiratory distress syndrome via suppressing of HMGB1/TLR9 pathways and neutrophils extracellular traps formation. Int immunopharmacology. (2022) 108:108730. doi: 10.1016/j.intimp.2022.108730, PMID: 35354111

[77] Feng X Chen W Ni X Little PJ Xu S Tang L. Metformin, macrophage dysfunction and atherosclerosis. Front Immunol. (2021) 12:682853. doi: 10.3389/fimmu.2021.682853, PMID: 34163481 PMC8215340

[78] Li J Xia Y Sun B Zheng N Li Y Pang X. Neutrophil extracellular traps induced by the hypoxic microenvironment in gastric cancer augment tumour growth. Cell communication signaling: CCS. (2023) 21:86. doi: 10.1186/s12964-023-01112-5, PMID: 37127629 PMC10152773

[79] Ye S Li S Ma Y Hu D Xiao F. Curcumin hinders PBDE - 47-induced neutrophil extracellular traps release via Nrf2-associated ROS inhibition. Ecotoxicology Environ Safety. (2021) 225:112779. doi: 10.1016/j.ecoenv.2021.112779, PMID: 34530259

[80] Zhang S Zhang Q Wang F Guo X Liu T Zhao Y. Hydroxychloroquine inhibiting neutrophil extracellular trap formation alleviates hepatic ischemia/reperfusion injury by blocking TLR9 in mice. Clin Immunol (Orlando Fla). (2020) 216:108461. doi: 10.1016/j.clim.2020.108461, PMID: 32437924

[81] Lingqing Y Chendong Ma Lei W Wang M Li Z Zhang L. Mechanism of regulation of NETs by glycyrrhizin to inhibit cell pyrodeath and relieve lung injury in sepsis. Chin J Emergency Med. (2024) 02):179–85. doi: 10.3760/cma.j.issn.1671-0282.2024.02.007

[82] Martinez NE Zimmermann TJ Goosmann C Alexander T Hedberg C Ziegler S. Tetrahydroisoquinolines: new inhibitors of neutrophil extracellular trap (NET) formation. Chembiochem: Eur J Chem Biol. (2017) 18:888–93. doi: 10.1002/cbic.201600650, PMID: 28240414

[83] Liu C Liu R Wang B Lian J Yao Y Sun H. Blocking IL - 17A enhances tumor response to anti-PD-1 immunotherapy in microsatellite stable colorectal cancer. J immunotherapy Cancer. (2021) 9(1):e001895. doi: 10.1136/jitc-2020-001895, PMID: 33462141 PMC7813395

[84] Scalzo-Inguanti K Monaghan K Edwards K Herzog E Mirosa D Hardy M. A neutralizing anti-G-CSFR antibody blocks G-CSF-induced neutrophilia without inducing neutropenia in nonhuman primates. J leukocyte Biol. (2017) 102:537–49. doi: 10.1189/jlb.5A1116-489R, PMID: 28515226

[85] Liu LN Chen C Xin WJ Li Q Han C Hua ZC. The oncolytic bacteria-mediated delivery system of CCDC25 nucleic acid drug inhibits neutrophil extracellular traps induced tumor metastasis. J nanobiotechnology. (2024) 22:69. doi: 10.1186/s12951-024-02335-5, PMID: 38369519 PMC10875894

[86] Hu JJ Liu X Xia S Zhang Z Zhang Y Zhao J. FDA-approved disulfiram inhibits pyroptosis by blocking gasdermin D pore formation. Nat Immunol. (2020) 21:736–45. doi: 10.1038/s41590-020-0669-6, PMID: 32367036 PMC7316630

[87] Silva CMS Wanderley CWS Veras FP Sonego F Nascimento DC Gonçalves AV. Gasdermin D inhibition prevents multiple organ dysfunction during sepsis by blocking NET formation. Blood. (2021) 138:2702–13. doi: 10.1182/blood.2021011525, PMID: 34407544 PMC8703366

[88] Yang S Feng Y Chen L Wang Z Chen J Ni Q. Disulfiram accelerates diabetic foot ulcer healing by blocking NET formation via suppressing the NLRP3/Caspase-1/GSDMD pathway. Trans research: J Lab Clin Med. (2023) 254:115–27. doi: 10.1016/j.trsl.2022.10.008, PMID: 36336332

[89] Metzler KD Fuchs TA Nauseef WM Reumaux D Roesler J Schulze I. Myeloperoxidase is required for neutrophil extracellular trap formation: implications for innate immunity. Blood. (2011) 117:953–9. doi: 10.1182/blood-2010-06-290171, PMID: 20974672 PMC3035083

[90] Sprenkeler EGG Zandstra J van Kleef ND Goetschalckx I Verstegen B Aarts CEM. S100A8/A9 is a marker for the release of neutrophil extracellular traps and induces neutrophil activation. Cells. (2022) 11(2):236. doi: 10.3390/cells11020236, PMID: 35053354 PMC8773660

[91] Li P Li M Lindberg MR Kennett MJ Xiong N Wang Y. PAD4 is essential for antibacterial innate immunity mediated by neutrophil extracellular traps. J Exp Med. (2010) 207:1853–62. doi: 10.1084/jem.20100239, PMID: 20733033 PMC2931169

[92] De Meo ML Shahzad MH Spicer JD. Visualizing NETosis using a novel neutrophil extracellular trap-specific marker. Methods Mol Biol (Clifton NJ). (2023) 2614:71–80. doi: 10.1007/978-1-0716-2914-7_5, PMID: 36587119

[93] Tilley DO Abuabed U Zimny Arndt U Schmid M Florian S Jungblut PR. Histone H3 clipping is a novel signature of human neutrophil extracellular traps. eLife. (2022) 11. doi: 10.7554/eLife.68283, PMID: 36282064 PMC9665850

[94] Tin W Xiao C Sun K Zhao Y Xie M Zheng J. TRIM8 as a predictor for prognosis in childhood acute lymphoblastic leukemia based on a signature of neutrophil extracellular traps. Front Oncol. (2024) 14:1427776. doi: 10.3389/fonc.2024.1427776, PMID: 39224802 PMC11366590

[95] Jia Y Jia R Taledaohan A Wang Y Wang Y. Structure-activity relationship of PAD4 inhibitors and their role in tumor immunotherapy. Pharmaceutics. (2024) 16(3):335. doi: 10.3390/pharmaceutics16030335, PMID: 38543229 PMC10975299

[96] Wang B Su X Zhang B Pan S. GSK484, an inhibitor of peptidyl arginine deiminase 4, increases the radiosensitivity of colorectal cancer and inhibits neutrophil extracellular traps. J Gene Med. (2023) 25:e3530. doi: 10.1002/jgm.3530, PMID: 37203323

